# When treating medial patellar luxation in dogs is a block trochleoplasty superior over a wedge trochleoplasty?

**DOI:** 10.18849/ve.v7i3.517

**Published:** 2022-07-15

**Authors:** Maria Norell Candetoft+

**Keywords:** BLOCK, RECESSION TROCHLEOPLASTY, DOGS, MEDIAL PATELLA LUXATION, TROCHLEAR BLOCK RECESSION, TROCHLEAR WEDGE RECESSION, WEDGE RECESSION TROCHLEOPLASTY

## Abstract

PICO question

As part of the surgical correction for medial patellar luxation in dogs, which procedure results in a better outcome for the patient: block or wedge recession trochleoplasty?

Clinical bottom line

Category of research question

Treatment

The number and type of study designs reviewed

Three studies satisfied the inclusion criteria for answering the PICO; one cadaver study, one retrospective observational study and one clinical case series

Strength of evidence

Weak

Outcomes reported

Postoperative complications including reluxation rates.

Ex vivo: Trochlear groove depth, patella articular contact, percentage of recessed trochlear surface area, resistance to medial patella luxation

Conclusion

There is only weak evidence to support block recession trochleoplasty over wedge recession trochleoplasty as part of the surgical correction for medial patella luxation in dogs. Both procedures are associated with a good clinical outcome. There are some proposed benefits to trochlear block recession made from an ex vivo study comparing the two procedures. These include an increased patellar volume under the trochlear ridges when the stifle is extended. The articular contact and recessed trochlear surface area were also increased in the trochlear block recession group when compared to trochlear wedge recession. However, the clinical relevance of these perceived benefits remains unproven. In practice, and until prospective randomised controlled trials are carried out, veterinary surgeon preference and previous experience remain relevant factors in choosing which procedure to perform

How to apply this evidence in practice

The application of evidence into practice should take into account multiple factors, not limited to: individual clinical expertise, patient’s circumstances and owners’ values, country, location or clinic where you work, the individual case in front of you, the availability of therapies and resources.

Knowledge Summaries are a resource to help reinforce or inform decision making. They do not override the responsibility or judgement of the practitioner to do what is best for the animal in their care.

## Clinical scenario

You have been working for a long time in a large veterinary hospital and over the years performed many block recession trochleoplasties as part of the surgical treatment of medial patellar luxation, and you have been very satisfied with the postoperative results. Now you have recently been employed by another veterinary hospital where all veterinary surgeons perform wedge recession trochleoplasty and they want you to switch your method since that they think that wedge recession is superior. You decide to read about the topic to learn more regarding the evidence between the two methods. To do a block or a wedge, that is the question.

## The evidence

The literature search uncovered only three papers that addressed the PICO question and fitted the inclusion and exclusion criteria applied. The three studies described the outcome of wedge recession trochleoplasty and block recession trochleoplasty; one study in cadavers (Johnson et al., 2001), one retrospective observational study (Rossanese et al., 2019) and one case series study (Ballatori et al., 2005). The number of cases included in the cadaver study by Johnson et al. (2001) was low, 12 cases with 24 stifles, and only four cases with eight stifles were included in the case series study. The latter study by Ballatori et al. (2005), was included despite the fact that it had both lateral and medial patellar luxations, although only the results applying to the medial patellar luxation (two dogs) were included. The study that included most animals was Rossanese et al. (2019) with 87 cases and a total of 100 stifles

In the study by Johnson et al. (2001) the difference in patellar depth, patellar articular contact, percentage of recessed trochlear surface area, and resistance to medial patellar luxation in stifles treated with trochlear block recession or trochlear wedge recession were described. In the specimens, the block technique was superior to the wedge technique as it had a deeper proximal patellar depth, a greater patellar articular contact in the extended stifle (P< 0.01), a larger percentage of recessed trochlear surface and a greater resistance to medial patellar luxation. Since the study was performed on cadavers with specimens mounted in a position device, it is difficult to interpret the results in live animals. Also, all dogs were large breed dogs, and it is unclear whether or not the results can be applied also to small breed dogs.

In the study by Rossanese et al. (2019) femoral trochleoplasty was performed in 90 stifles and included a trochlear wedge recession in 68 stifles (76%) and a trochlear block recession in 22 stifles (24%). Results from this study showed no significant difference in complication rates between the trochlear wedge technique and the trochlear block technique. The type of trochleoplasty performed (block vs wedge) was not associated with the occurrence of postoperative complications.

Valid conclusions may not be drawn from the study by Ballatori et al. (2005) as only four dogs and eight stifles were included, and two dogs had medial patellar luxation and two dogs had lateral patellar luxation.

## Summary of the evidence

**Johnson et al. (2001):** 

Population:	Normal, large-breed canine cadavers. The dogs were euthanised for reasons unrelated to the study and were determined to be free of stifle disease via orthopaedic examination and radiographs. Mean weight was 31.9 kg (25.0–38.6 kg).
Sample size:	24 stifles from 12 dogs.
Intervention details:	Specimens consisting of femur, tibia and intact stifle joint were removed bilaterally from each cadaver. Soft tissues were dissected free, leaving only the stifle joint capsule and ligaments intact. The quadriceps tendon of insertion on the patella was transected 1.5 cm proximal to the patella. Bilateral pelvic limb specimens were mounted on two identical position devices so that the legs could be examined and tested in standardised methods. To simulate a shallow trochlea, the trochlear ridges of each specimen were reduced using a high-speed drill and burr. A plastic guiding template was used to make the trochlea ridges at the same (low) height on each specimen. The joint capsule was opened laterally (to mimic a stretched lateral joint capsule) and a mechanic arm applied 40° of internal tibial rotation in both flexion and extension. The medially internal rotation made all of the patellas to luxate medially within 40° of internal tibial rotation in each trial. Trochlear block recession (TBR) and trochlear wedge recession (TWR) was performed on opposite stifles of paired specimens, selection of right or left leg in each pair was managed by flipping a coin. Depth and position of TWR were standardised between specimens using a custom-made template that directed the saw blade in the same way in all the trochleas. The position of the TBR was also standardised between specimens based on anatomic landmarks. Each specimen was evaluated before and after trochleoplasty (TBR or TWR) with computed tomography (CT) and biomechanical testing with the stifle held in extension (148° to evaluate the patella within the proximal trochlea) and in flexion (113° to evaluate the patella within the central trochlea). The depth of the trochlea in the central and proximal portion was measured as well as the patellar coverage percent. The patellar articular contact with the recessed trochlea was also determined postoperatively. The percentage of recessed trochlear surface area was determined for each specimen.
Study design:	In vitro CT and biomechanical evaluation using a cadaver model.
Outcome studied:	To compare TBR to TWR with regards to patellar depth (percentage of patellar volume under the trochlear ridges), patellar articular contact, percentage of recessed trochlear surface area, and resistance to patellar luxation.
Main Findings (relevant to PICO question)	No significance in the depth of trochlear recession existed between groups. Postoperative patellar depth in the extended stifle position was significantly greater in the TBR group compared with the TWR group. In the extended stifle position, postoperative patellar articular contact was significantly greater in the TBR group compared with the TWR group. Postoperative recessed trochlear surface area was significantly greater in the TBR group (76.5%) compared with the TWR group (49.9%). Postoperatively patellar luxation did not occur in any specimen in the flexed stifle but in the extended stifle (with the leg held in 40° internal rotation) the patella luxated in 8% (1/12) after TBR and in 42% (5/12) after TWR. The difference was not statistically significant (P = 0.06).
Limitations:	Although more versatile, CT is a less sensitive imaging modality for visualising articular cartilage compared with magnetic resonance imaging (MRI). However, to investigate the groove depth of the trochleoplasty it is easier to make measurements from CT reconstruction / multiplanar image manipulation compared to MRI. This was a cadaver study so we do not know how to correctly interpret the data in live animals. The authors did not attempt to approximate physiologic forces placed on the patella by the quadriceps muscle group. The model also does not account for tibial or femoral torsional deformities commonly seen in dogs with patellar luxation. The test was only performed in specimens from large breed dogs so we do not know if the results would be the same for small breed dogs.

**Rossanese et al. (2019):** 

Population:	Dogs <20 kg surgically treated for medial patellar luxation between 2011–2016. The preoperative luxation grade was classified as Grade I in one stifle Grade II in 51 stifles, Grade III in 42 stifles and Grade IV in six stifles. Median weight 8.2 kg (total range 1.2–20.00 kg).
Sample size:	87 dogs met the inclusion criteria and a total of 100 surgical procedures for medial patellar luxation were performed.
Intervention details:	Surgery was performed by an experienced surgeon in 70 cases and by a resident under supervision in 30 cases. No dogs in the study required corrective osteotomy techniques in the distal femur. All surgical procedures included lateral tibial tuberosity transposition (TTT). Medial soft tissue release was performed in 41 stifles (41%). Lateral imbrication was performed in 81 stifles (81%). Femoral trochleoplasty was performed in 90 stifles (90%) and included a trochlear wedge recession in 68 stifles (76%) and a trochlear block recession in 22 stifles (24%). Medial surgical time was 75 minutes (total range 25–195 minutes).
Study design:	Retrospective observational study.
Outcome studied:	Complications in dogs weighing <20 kg surgically treated for medial patellar luxation and to determine risk factors associated with these complications.
Main Findings (relevant to PICO question)	37 stifle joints developed postoperative complications. 12 were considered minor (patellar reluxation Grade I, tibial tuberosity fracture, skin irritation), and 25 complications were considered major (patellar luxation Grade II, surgical site infection, wound dehiscence, removal of K-wires because of pin-fracture, pin-migration or seroma). Results relevant to the PICO question included four cases of Grade I patellar luxation that did not require reoperation, and two cases of Grade II patella luxation that required revision surgery. Results from the study showed no significant difference in complication rates between the trochlear wedge technique and the trochlear block technique.
Limitations:	Retrospective observational studies are considered to provide low-level scientific evidence. The data reported relies on the accuracy of the medical record entries. The article identified long-term complications by reviewing the referring veterinarian clinical records and it might be possible that some long-term complications were not noticed by the referring veterinarian. We do not know if the wedge trochleoplasty has a greater tendency to reluxate after several years, when the dog is older and has less muscle mass compared with the block trochleoplasty or vice versa, or if none of the techniques reluxates later on in life. The study did not address the progression of osteoarthritis. There were no standardised protocols to compare the different techniques. All of them performed transposition of the tibial tuberosity, but in some cases imbrication of the joint capsule and / or medial release of the joint capsule was also performed. Since the article only reviewed dogs less than 20 kg of body weight, we do not know if either of the two different trochleoplasty techniques are more or less suitable for heavier dogs. As surgical procedures cannot be clearly differentiated from the surgeons performing them, we do not know whether or not we are investigating the surgeons or the procedures.

**Ballatori et al. (2005):** 

Population:	Dogs with bilateral patellar luxation Grade I–IV brought to the department with a complaint about secondary lameness, between the years 2002–2004.
Sample size:	Eight stifles in four dogs. Two dogs had medial patellar luxation and two dogs had lateral patellar luxation.
Intervention details:	Radiographic examination was performed to exclude severe malformation of the femur and tibia. Two types of surgeries were performed in the same dog: trochlear block recession (TBR) in the right stifle and trochlear wedge recession (TWR) in the left stifle. Both procedures were performed in the same operation. Both of the dogs with medial patellar luxation also had tibial tubercle transposition (transposition of tibial tuberosity). The dogs had a postoperative period of physiotherapy with gradual return to normal activity in about 2 months. Follow-up period: 10–15 days postoperatively, 2–3 months postoperative and one case 12 months postoperatively.
Study design:	Clinical case series.
Outcome studied:	Immediate postoperative computed tomography (CT): Direct after the surgical procedure the stifles were examined with CT in ventrodorsal position with semi-flexed pelvic limbs. The CT images was used to estimate trochlear depth and patellar depth. A line between the trochlear ridges and the centre of the trochlear surface provided the measurement for the trochlear depth. The patellar depth was measured as the percentage of the entire patellar volume positioned under the trochlear ridges. Clinical evaluation 10–15 days postoperative: 10–15 days postoperative the Robert Jones bandage was removed and clinical articular evaluation was assessed. Clinical evaluation 2–3 months postoperative: 2–3 months after surgery the dogs were evaluated clinically regarding distribution of weight between the two hindlegs, tolerance during activity, muscular growth, patellar stability and pain. One dog was examined with CT 12 months after surgery.
Main Findings (relevant to PICO question)	CT findings: The CT showed correct autograft positioning and an adequate patellar lodging into the trochlear groove in all cases, independently of surgical technique used. In the central trochlea both TBR and TWR allowed the achievement of a good trochlear depth, but the patellar depth was greater in the knee with rectangular recession. The proximal trochlear sulcus was wider and deeper with TBR than with TWR. The distal part of the subchondral wedge reached deeper in the caudal portion of the femoral trochlea but the proximal part of the autograft wedge is entirely excavated in the cranial portion of the trochlea. Findings in the clinical evaluation: In the postoperative period no complications or relapses in any of the dogs were seen. 15 days after surgery the patients showed a fairly good ability to distribute weight on both operated limbs but there was more lameness and pain in articulations with TBR. The postoperative clinical picture 2–3 months after surgery was similar in both joints, with the difference that the patella was stable in the flexed position but in extended position a more lateromedial patellar instability was recognised in stifles treated with trochlear wedge recession.
Limitations:	Very small sample size. Case series which is lower in evidence hierarchy. The group was not homogenous: the dogs with medial patellar luxation were of different age (18 months and 7 years), different breeds (Pinscher and Springer Spaniel) the grade of patellar luxation was not the same (ranging from Grade I–IV) and it was not the same grade between the legs in the same patient. No observer standardised clinical examination was described, introducing observer bias. It does not say if the patellas luxated postoperatively, only that the patella was more unstable in a lateromedial direction in the trochlear wedge technique. No description of inclusion or exclusion criteria (except for radiographic examination for malformation of femur / tibia). No long-term follow-up was available to examine if any surgical method acquired less degenerative joint disease.

## Appraisal, application and reflection

Outcome as a measure of success might be difficult to objectively assess as it is influenced by many different factors. When surgically correcting a medial patellar luxation one seldom only corrects the trochlea but most often also amends the soft tissues (imbrication of the joint capsule, release incision of the retinaculum or desmotomi) in combination with longitudinal realignment of the tibial tuberosity relative to the trochlear groove, by performing a tibial tuberosity transposition (Arthurs & Langley-Hobbs, 2006). Thus, it is difficult to determine if a trochlear block recession is superior to a wedge recession technique as the two different surgical methods are not performed in isolation.Only three studies were identified addressing the PICO question; one biomechanical cadaver study, one retrospective observational study with 100 stifles and one case series including only two dogs. Thus, the evidence base for answering the query if block recession trochleoplasty is superior to wedge recession trochleoplasty when treating medial patellar luxation is indeed very limited.Of the three studies, the biomechanical cadaver study (Johnson et al., 2001) has a more impressive implementation with ex vivo testing of a stifle model that mimics patellar luxation, objective interobserver assessment and distinct variable measurements obtained from the computed tomography. The main limitation with the cadaver study, when using it to answer the PICO question, is that results might not be applicable to live animals. Also, long-term effects cannot be evaluated.The retrospective observational study by Rossanese et al. (2019) described complications following surgical correction of medial patellar luxation in dogs < 20 kg. In the study including 87 dogs and 100 stifles with different degree of patellar luxation, complications were recognised in 37 stifles; 12 minor and 25 major. The outcome relevant to the PICO question showed no significant difference in complication rates between trochlear wedge resection and trochlear block resection. One of the main limitations with this study, apart from it being a retrospective study, is that it lacks long-term follow-up. We do not know if any of the trochleoplasty techniques had worse or more favourable outcome after several years. Also, to use this article to answer the question whether or not one surgical method is superior to another is difficult because, there are no kinematics, no description of repeated clinical examinations, no owner questionnaires, or second-look arthroscopy.  However, a second look arthroscopy may not be of added value to the animal and thus not considered to be ethical.In addition, time records to perform each procedure are not published. It is therefore not possible to comment if one procedure is less time consuming. This would be an interesting factor to compare.Generally, retrospective case series are regarded low on the hierarchy of the evidence scale. In the case series by Ballatori et al. (2005), the number of dogs studied are by far too low to allow a meaningful comparison between the treatment methods as only two dogs had medial patellar luxation and also with different luxation degree in the individual hindleg.The difference between the two surgical methods judged by the use of computed tomography is a result of the geometry of the block and wedge. The articular width of the block and wedge are similar in the centre of the trochlea, but the block maintains the articular surface width along the entire length of the trochlea compared to the wedge, which tapers to a point proximally and distally. After trochlear wedge recession, as the stifle is extended and the patella moves proximally, the patella may articulate with the non-recessed proximal femoral trochlea instead of the recessed articular cartilage of the wedge, resulting in decreased patellar depth. The reduction in proximal patellar height could be essential in the clinical treatment of patellar luxation since the patella most often luxate in the proximal trochlea when the stifle is extended (Talcott et al., 2000, and Johnson et al., 2001).In dogs with medial patellar luxation, block trochlear recession results in a proximal deeper trochlear groove and a larger contact area between the proximal trochlea and patella, compared with the wedge trochlear recession. In the extended stifle, the patella lies deeper in the proximal part of the joint (Johnson et al., 2001; and Talcott et al., 2000). Since no long-term studies comparing the amount of degenerative changes in the stifle joint and re-luxation rates between the two methods exist, and since the scientific information that actually compares the methods are very limited, no conclusion can be made from the existing evidence regarding whether or not a trochlear block recession is superior to trochlear wedge recession technique, when treating medial patellar luxation in dogs.Since there are few reports comparing the two surgical methods, studies describing outcomes and complication rates with each technique might provide useful information when choosing between the two surgical methods: Slocum et al. (1982) performed trochlear wedge surgery and showed excellent results in 13/17 stifles and good results in the remaining cases. The follow-up period in that study was 12–29 months. Talcott et al. (2000) described 100 dogs after trochlear block surgery with positive short-term results: the joints were considered free of crepitus, limb function was improved and patellar stability achieved in the 6 week follow-up period. However, no long-term radiographic studies, histological analyses or second-look arthroscopy was performed in that study.  A retrospective case series by Gallegos et al. (2016) described bilateral wedge trochleoplasty in 50 small breed dogs (100 stifle joints) with medial patellar luxation. In the study, 5/50 dogs (10%) had reluxation (Grade I) none of the dogs showed clinical lameness postoperatively. The median follow-up time was 8 weeks. Arthurs & Langley-Hobbs (2006) reported retrospectively the clinical outcome in 109 dogs undergoing surgery because of lateral or medial patellar luxation. In the study, 74/107 (69%) had trochlear wedge recession and 8/107 (7%) had trochlear block recession. However, no comparison was made between the methods. In another retrospective study by Gibbons et al. (2006) trochlear wedge or trochlear block was performed to treat patellar luxation in 70 large breed dogs (>15 kg). As no comparisons were made between the two methods, no conclusions regarding the superiority of either method can be drawn. Cashmore et al. (2014) reported a retrospective study regarding complications and risk factors associated with surgical correction of medial patellar luxation in 124 dogs. Major complications (implant associated, patellar luxation and persisting lameness, patellar tendon rupture etc.) occurred in 24/124 (19%) of dogs. Although no comparisons were made between methods, a case of trochlear wedge displacement was reported. In a pilot study (Blackford-Winders et al., 2021) of 10 dogs, where the trochlear block recession technique was performed, the block autograft fractured in three cases. In a case report by Ellis & House (2021) the trochlear block migrated distally 7 days postoperatively. In another case report (Chase & Farrell, 2010) a fracture of the lateral trochlear ridge following trochlear block recession was described.One plausible reason for recurrent postoperative patellar luxation may, at least in part, be caused by inadequate appreciation of the underlying skeletal deformity and subsequent selection and application of corrective surgery. Accurately measuring anatomic conformational abnormalities, for example identifying an extensive varus deformity or torsion in the distal femur, to better understand the deformities and subsequently better tailor corrective surgery by performing different kind of osteotomies than trochleoplasties, of the distal femur or proximal tibia, may result in lower frequency of reluxation.In conclusion, further studies are needed to evaluate both short-term and long-term clinical outcome in small breed dogs with medial patellar luxation undergoing either trochlear wedge recession or trochlear block recession. On one hand, the block technique might be considered to be more physiologically or anatomically appropriate creating a deeper trochlea proximally in the joint and a more profound femoropatellar contact. On the other hand, the wedge technique is less invasive and thus conceivably associated with lower risk of complications. Whether or not the degree of subsequent osteoarthrosis might be influenced by the choice of surgical technique is of major importance for the individual dog. The preference and previous experiences of the veterinary surgeon are relevant issues when choosing which operation to perform until randomised and controlled trials in live animals and in comparable groups are performed.

## Methodology Section

**Search Strategy:** 

Databases searched and dates covered:	CAB Abstracts on the OVID interface 1973–2021 week 46 PubMed accessed via the NCBI website 1920–Nov 2021
Search strategy:	CAB Abstracts: (dog or dogs or canine or canines or canis or bitch or bitches or puppy or puppies).mp. or exp dogs/ or exp bitches/ or exp puppies/ or exp canidae/ or exp canis/ (lux* or MPL or dislocat*).mp. or exp dislocation/ ((trochle* or sulcoplas*) and (wedge or block or recession)).mp. 1 and 2 and 3 PubMed: dog or canine or bitch or puppy luxation or MPL or dislocation (trochleoplasty or sulcoplasty or trochlear) and (wedge or block or recession) 1 and 2 and 3
Dates searches performed:	22 Nov 2021

**Exclusion / Inclusion Criteria:** 

Exclusion:	Non-English language.
Inclusion:	Articles concerning canine stifles with medial patellar luxation that have undergone either trochlear block recession or trochlear wedge recession.

**Search Outcome:** 

Database	Number of results	Excluded – Wrong species	Excluded – Not in English	Excluded – Not relevant to the PICO question	Total relevant papers
CAB Abstracts	48	2	4	40	2
PubMed	20	1	0	18	1
Total relevant papers when duplicates removed					3

## Conflict of interest

The authors declare no conflicts of interest.
